# The Correlation between Rates of Cancer and Autism: An Exploratory Ecological Investigation

**DOI:** 10.1371/journal.pone.0009372

**Published:** 2010-02-23

**Authors:** Hung-Teh Kao, Stephen L. Buka, Karl T. Kelsey, David F. Gruber, Barbara Porton

**Affiliations:** 1 Department of Psychiatry and Human Behavior, Brown University, Providence, Rhode Island, United States of America; 2 Department of Community Health, Brown University, Providence, Rhode Island, United States of America; 3 Department of Natural Sciences, Baruch College and The Graduate Center, City University of New York, New York, New York, United States of America; University of Texas MD Anderson Cancer Center, United States of America

## Abstract

**Background:**

Autism is associated with high rates of genomic aberrations, including chromosomal rearrangements and *de novo* copy-number variations. These observations are reminiscent of cancer, a disease where genomic rearrangements also play a role. We undertook a correlative epidemiological study to explore the possibility that shared risk factors might exist for autism and specific types of cancer.

**Methodology/Principal Findings:**

To determine if significant correlations exist between the prevalence of autism and the incidence of cancer, we obtained and analyzed state-wide data reported by age and gender throughout the United States. Autism data were obtained from the U.S. Department of Education via the Individuals with Disabilities Education Act (IDEA) (2000–2007, reported annually by age group) and cancer incidence data were obtained from the Centers for Disease Control and Prevention (CDC) (1999–2005). IDEA data were further subdivided depending on the method used to diagnose autism (DSM IV or the Code of Federal Regulations, using strict or expanded criteria). Spearman rank correlations were calculated for all possible pairwise combinations of annual autism rates and the incidence of specific cancers. Following this, Bonferroni's correction was applied to significance values. Two independent methods for determining an overall combined *p*-value based on dependent correlations were obtained for each set of calculations. High correlations were found between autism rates and the incidence of *in situ* breast cancer (*p*≤10^−10^, modified inverse chi square, n = 16) using data from states that adhere strictly to the Code of Federal Regulations for diagnosing autism. By contrast, few significant correlations were observed between autism prevalence and the incidence of 23 other female and 22 male cancers.

**Conclusions:**

These findings suggest that there may be an association between autism and specific forms of cancer.

## Introduction

Autism is a pervasive developmental disorder characterized by severe impairments in social skills, language and communication, as well as behavioral disturbances. There is growing public awareness of autism because rates of this disorder are thought to be rising [1]. The etiology of autism is still unknown and clues as to its cause are urgently needed.

Previous studies have reported that children with autism possess a higher number of genetic aberrations, including higher levels of chromosomal rearrangements [2] and copy number variations [3], [4], [5], [6], [7]. These studies raise the possibility that there may be correlations to cancer, a disease in which chromosomal aberrations are known to play a role. Here, we report a study in which the incidence of cancer is compared to the prevalence of autism.

Beginning in 1975, the Individuals with Disabilities Education Act (IDEA) was passed, mandating that states report the number of children who undergo special education, subdivided according to a specified disability. In 1991, autism was added as a separate category by which states must report child count numbers. The IDEA database represents the sole national source of autism prevalence statistics in the U.S. Despite limitations, the IDEA data are the best available for estimates of autism prevalence in the U.S., and recent improvements have been made to this data system. For example, the methods by which states formally diagnose children with autism have been analyzed, and those states adhering to uniform criteria were identified [8]. Cancer statistics, by contrast, are collected with rigor, and the diagnosis is rarely in dispute and methods for determining cancer diagnosis are firmly established. Here, we present an analyses using both cancer and autism databases, incorporating information about state-level differences in autism diagnosis [8].

## Results

### State-Level Correlations of Autism Prevalence with Incidence of All Cancers

**Figure 1:**
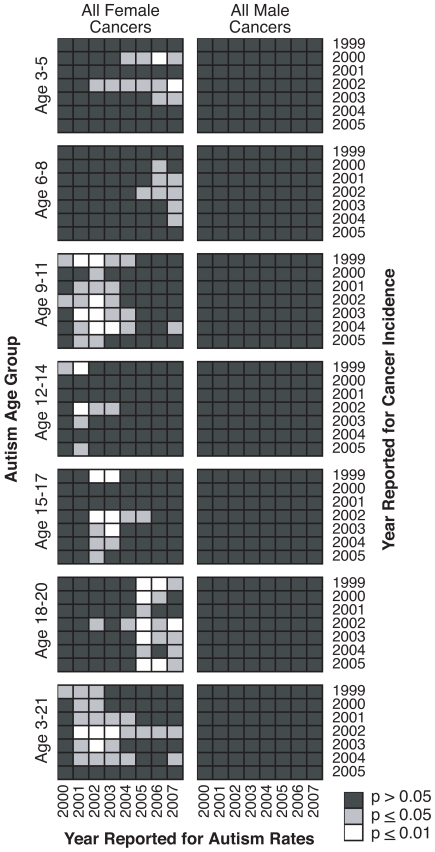
Spearman rank correlations between annual cancer incidence and autism prevalence. Pairwise correlations were conducted between the annual incidence of adult cancers (all cancers combined) and the prevalence of autism. For each age group, 56 possible pairwise correlations depending on year were determined. For each year that state cancer incidence (from the CDC) and autism prevalence (from the IDEA) were reported, a two-tailed Spearman Rank correlation coefficient was determined. Significance was adjusted using Bonferroni's correction [10] and shaded as indicated to facilitate visual inspection of the results. The CDC consolidates 24 anatomic sites for all female cancers and 22 anatomic sites for all male cancers.

As depicted in Figure 1, we present Spearman rank correlations at the state level between autism prevalence (according to age groups and year reported) and cancer incidence (for the specific cancer type or group of cancers by gender and year). All possible combinations of autism and cancer data were correlated to avoid Type 1 bias, and the results were tabulated on a grid depicting the years for which autism or cancer data were reported (Fig. 1).

Autism prevalence data before the year 2000 were omitted from these analyses because: 1) Data for ages 3–5 are unavailable prior to 2000; and 2) the latest diagnostic criteria for autism, DSM-IV TR, was introduced in 2000.

There are 56 different combinations by which autism prevalence (for a specific age group) and cancer incidence may be compared (Fig. 1). Some combinations yield a nominally significant correlation while others do not. Multiple correlations can introduce Type I error (acceptance of a false correlation), a common problem when relationships between two types of biological measurements are extrapolated [9]. Therefore, all the *p*-values were adjusted using the Bonferroni method of correction, a conservative technique for reducing Type I error. Thus, all calculated *p*-values were multiplied by 56 to yield an adjusted *p*-value not to exceed 1 (i.e. the *p*-value was adjusted to 1 if the Bonferroni correction yielded a value above 1) [10]. Using this approach, Bonferroni-adjusted *p*-values <0.05 are considered statistically significant (which corresponds to an initial, unadjusted *p*-value <0.0009).

The correlations with autism prevalence conducted in Fig. 1 utilized annual state-level incidence of all cancers according to gender. A pattern of significant correlations emerges from the data between all female cancers and autism, but not between all male cancers and autism.

We sought a method for reporting the Spearman rank correlations between annual cancer incidence and autism prevalence as a group, incorporating all Bonferroni-adjusted *p*-values (both nonsignificant and significant) to produce a combined overall *p*-value (summarized in Table 1). One possibility is to record the percentage of nominally significant correlations (out of 56 correlations conducted per comparison, using the adjusted *p*-values). Another possibility is to use Fisher's inverse chi-square method [11], a well-established procedure for combining *p*-values obtained from independent observations, significant or otherwise. However, each individual *p*-value comes from observations that are not actually independent from one another, as will be described further in the Discussion. Two methods for combining a group of dependent *p*-values were used: a modified version of Fisher's chi-square method that takes into account the relationship among the *p*-values [12], and an improved Bonferroni procedure that rank *p*-values from the lowest to the highest values [13]. As shown in Table 1, the overall Bonferroni-adjusted *p*-value was nominally significant for correlations between autism prevalence and the incidence of all female but not male cancers. The *p*-values determined using Fisher's inverse chi-square method are very low, likely because the underlying assumption when using this method is that the *p*-values come from independent observations. Since this assumption is unlikely, the methods described by Brown or Simes are more appropriate for this analysis, and are reported in the subsequent Tables.

**Table 1:** Correlations Between the Annual Incidence of All Adult Cancers and Autism Prevalence.

	Female Cancer Incidence correlated with Autism Prevalence				Male Cancer Incidence correlated with Autism Prevalence			
AGE	% *p*<0.05†	Fisher's P	Brown's P	Simes' P	% *p*<0.05†	Fisher's P	Brown's P	Simes' P
3–5	21.4	**7.3×10^−8^**	0.040	**0.003**	0	1	1	0.047
6–8	14.3	**5.8×10^−6^**	0.136	**0.005**	0	1	1	0.163
9–11	42.9	**1.6×10^−20^**	**6.9×10^−7^**	**0.001**	0	1	1	0.126
12–14	12.5	**1.6×10^−7^**	0.051	**0.002**	0	1	1	0.094
15–17	19.6	**6.8×10^−6^**	0.142	**0.002**	0	1	1	0.166
18–20	33.9	**4.3×10^−11^**	**0.004**	**0.0005**	0	1	1	0.064
**3–21**	42.9	**3.3×10^−22^**	**1×10^−7^**	**0.001**	0	1	1	0.110

† % *p*<0.05 refers the to percentage of correlations out of the total number (56) that reach a nominal significance of *p*<0.05 (Bonferroni-adjusted).

All possible combinations of pairwise correlations were performed between annual cancer incidence (all cancers combined) in females and males and the estimated prevalence of autism (as in Fig. 1). Each set of comparisons (autism vs cancer for a specific autism age group) consists of 56 correlations. Four ways of presenting significance are tabulated: 1) the percentage of correlations in which *p*<0.05 (% *p*<0.05; Bonferroni-adjusted); 2) an overall *p*-value using Fisher's inverse chi-square method (Fisher's P) [11]; 3) a modified inverse chi-square for dependent *p*-values (Brown's P) [12]; and 4) a modified Bonferroni procedure to obtain an overall *p*-value (Simes' P) [13]. Nominally significant values (*p*≤0.01) are bolded.

**Figure 2:**
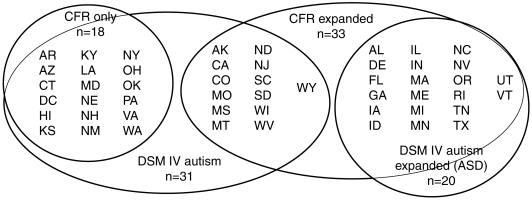
Autism diagnostic criteria used by states. The number and identity (by postal state abbreviation) of states that adhere to strict wording in the CFR (CFR only) or expanded criteria (CFR expanded) to diagnose autism are indicated. The diagnosis of autism by CFR is included within the DSM-IV-TR definition, and those states that use the DSM-IV-TR theme are shown (DSM IV autism). States that expand their criteria to include autism spectrum disorder (ASD) represent the fourth criteria used (DSM IV expanded (ASD)).

A thorough analysis of each state's approach to diagnosing autism was recently published [8], thus allowing us to categorize states according to diagnostic method. Autism prevalence data obtained under the IDEA does not depend on DSM-IV-TR criteria (although it may for specific states), but rather, depends on the Code of Federal Regulations (CFR). Seventeen states and the District of Columbia apply a strict wording of the CFR to categorize children as being disabled by autism. The remaining states apply expanded criteria, including DSM-IV-TR or a broader definition to include all autism spectrum disorders. Four subdivisions of states (Fig. 2), were used to derive an overall *p*-value (Table 2). Significance depends on both effect size and sample size, and by lowering sample size, significance is reduced. Despite this potential drawback to the analysis, nominal significance was still observed between autism prevalence and the incidence of all female cancers combined.

**Table 2:** Correlations Between the Annual Incidence of All Adult Cancers Combined and Autism Prevalence Subdivided by Method of Diagnosis.

		ALL		Expanded Criteria (CFR)		Expanded Criteria (DSM-IV)		Autism (DSM-IV)		CFR	
	Method for Combining *p*-values	P	N	P	N	P	N	P	N	P	N
**All Female Cancers**	Brown	**1.0×10^−7^**	46	0.395	31	0.991	19	1	27	1	16
	Simes	**0.001**	46	0.004	31	**0.010**	19	0.062	27	0.164	16
**All Male Cancers**	Brown	1	46	1	32	1	19	1	27	1	16
	Simes	0.110	46	0.170	32	0.136	19	0.529	27	0.767	16

Pairwise correlations were performed as described in Table 1 using two methods, Brown and Simes [12], [13], for combining dependent *p*-values. Autism prevalence data (ages 3–21) were obtained from groups of states selected on the basis of their criteria for diagnosing autism in all states or states subdivided by 4 groups of criteria (Fig. 2). P represents combined *p*-values for the Spearman correlations and bolded if P≤0.01. N represents the median number of states for which both autism and cancer data were available for analyses. Data are similar when the Pearson Correlation Coefficient is used (Table S1).

### Correlations between Autism Prevalence and the Incidence of Specific Female and Male Cancers

The same types of analyses were applied to 24 specific cancers for females and 22 cancers for males (Tables 3, Table 4 Table 5 and Table 6). Using Brown's method for combining *p*-values and the most restrictive diagnostic classification, CFR, significant correlations with autism prevalence was observed with the incidence of only one cancer, breast cancer *in situ* (*p*<10^−10^; N = 16, Table 3). All other correlations between autism prevalence (using the CFR classification) and the other female cancers (Table 3) or male cancers (Table 5) were nonsignificant using Brown's method for combining *p*-values. Simes' method for combining *p*-values is less stringent, and other nominally significant correlations emerge using this test (Tables 4 & 6). Uterine cancer (Corpus and Uterus, NOS) displayed significant correlation with autism prevalence regardless of the diagnostic criteria used by state (Table 4). The Spearman rank correlation generally provided similar results when compared to the Pearson product moment coefficient (Tables S1, Table S2, Table S3, Table S4 and Table S5).

**Table 3:** Correlations Between the Annual Incidence of Specific Female Adult Cancers and Autism Prevalence Subdivided by Method of Diagnosis, using Brown's P-value Method.

	ALL		Expanded Criteria (CFR)		Expanded Criteria (DSM-IV)		Autism (DSM-IV)		CFR	
	P	N	P	N	P	N	P	N	P	N
Brain and Other Nervous System	1	44	1	29	1	19	1	25	1	15
Breast, invasive	0.108	46	0.914	30	0.881	19	1	27	1	16
Breast, in situ	**1.5×10^−18^**	46	1	30	1	19	0.861	27	**7.5×10^−12^**	16
Cervix Uteri	1	46	1	30	1	19	1	27	1	16
Colon and Rectum	1	46	1	30	1	19	1	27	1	16
Corpus and Uterus, NOS	**2.3×10^−12^**	46	1	30	1	19	0.638	27	0.990	16
Esophagus	1	34	1	22	1	15	1	20	0.979	13
Hodgkin Lymphoma	1	37	1	24	1	16	1	21	1	14
Kidney and Renal Pelvis	1	46	1	30	1	19	1	27	1	16
Larynx	1	32	1	21	1	14	1	18	1	11
Leukemias	1	46	1	30	1	19	1	27	1	16
Liver and Intrahepatic Bile Duct	1	38	1	24	1	16	1	22	1	14
Lung and Bronchus	1	46	1	30	1	19	1	27	1	16
Melanomas of the Skin	1	46	1	30	1	19	1	27	1	16
Mesothelioma	1	13	1	9	1	6	1	7	1	4
Myeloma	1	43	1	28	1	18	1	25	1	16
Non-Hodgkin Lymphoma	1	46	1	30	1	19	1	27	1	16
Oral Cavity and Pharynx	1	45	1	29	1	19	1	26	1	16
Ovary	1	46	1	30	1	19	1	27	1	16
Pancreas	1	46	1	30	1	19	0.996	27	1	16
Stomach	1	43	1	27	1	18	1	25	1	16
Thyroid	1	46	1	30	1	19	1	27	1	16
Urinary Bladder	0.011	46	1	30	1	19	1	27	1	16

Pairwise correlations were performed, as described in Table 1, between state-level annual incidence for specific female cancers and autism prevalence (ages 3–21) from states selected on the basis of their criteria for diagnosing autism (Fig. 2). P represents combined *p*-values for Spearman correlations using Brown's method and bolded if P≤0.01. N represents the median number of states for which both autism and cancer data were available for analyses. Kaposi's sarcoma is omitted because there was insufficient data to conduct the analyses. Data are similar when the Pearson Correlation Coefficient is used (Table S2).

**Table 4 pone-0009372-t004:** Correlations Between the Annual Incidence of Specific Female Adult Cancers and Autism Prevalence Subdivided by Method of Diagnosis, using Simes' P-value Method.

	ALL		Expanded Criteria (CFR)		Expanded Criteria (DSM-IV)		Autism (DSM-IV)		CFR	
	P	N	P	N	P	N	P	N	P	N
Brain and Other Nervous System	0.795	44	0.613	29	0.273	19	0.911	25	0.982	15
Breast, invasive	**0.002**	46	**0.005**	30	**0.003**	19	0.057	27	0.238	16
Breast, *in situ*	**2.2×10^−5^**	46	**0.006**	30	0.032	19	**0.002**	27	**0.001**	16
Cervix Uteri	0.064	46	0.843	30	0.141	19	0.198	27	0.015	16
Colon and Rectum	0.174	46	0.740	30	0.535	19	0.404	27	0.633	16
Corpus and Uterus, NOS	**0.0004**	46	0.015	30	0.032	19	**0.004**	27	**0.010**	16
Esophagus	**0.006**	34	0.193	22	0.407	15	0.036	20	**0.003**	13
Hodgkin Lymphoma	**0.004**	37	0.085	24	0.497	16	0.027	21	0.053	14
Kidney and Renal Pelvis	0.719	46	0.460	30	0.817	19	0.968	27	0.928	16
Larynx	0.034	32	0.158	21	0.479	14	0.242	18	0.322	11
Leukemias	0.506	46	0.977	30	0.852	19	0.192	27	0.478	16
Liver and Intrahepatic Bile Duct	0.860	38	0.981	24	0.901	16	0.999	22	0.999	14
Lung and Bronchus	0.102	46	0.087	30	0.063	19	0.966	27	0.981	16
Melanomas of the Skin	0.108	46	0.496	30	0.588	19	0.752	27	0.410	16
Mesothelioma	0.479	13	0.106	9	0.102	6	0.996	7	NA	4
Myeloma	0.768	43	0.682	28	0.923	18	0.997	25	0.976	16
Non-Hodgkin Lymphoma	0.013	46	0.012	30	0.068	19	0.261	27	0.740	16
Oral Cavity and Pharynx	0.016	45	0.070	29	0.290	19	0.100	26	0.301	16
Ovary	0.689	46	0.983	30	0.683	19	0.961	27	0.813	16
Pancreas	0.022	46	0.038	30	0.854	19	**0.001**	27	0.030	16
Stomach	0.065	43	0.150	27	0.992	18	0.069	25	0.228	16
Thyroid	0.994	46	0.992	30	0.996	19	0.774	27	0.533	16
Urinary Bladder	**0.002**	46	0.025	30	0.023	19	0.110	27	0.020	16

Pairwise correlations were performed, as described in Table 1, between state-level annual incidence for specific female cancers and autism prevalence (ages 3–21) from states selected on the basis of their criteria for diagnosing autism (Fig. 2). P represents combined *p*-values for Spearman correlations using Simes' method and bolded if P≤0.01. N represents the median number of states for which both autism and cancer data were available for analyses. Kaposi's sarcoma is omitted because there was insufficient data to conduct the analyses. Data are similar when the Pearson Correlation Coefficient is used (Table S3).

**Table 5 pone-0009372-t005:** Correlations between the Annual Incidence of Specific Male Adult Cancers and Autism Prevalence Subdivided by Method of Diagnosis, using Brown's P-value Method.

	ALL		Expanded Criteria (CFR)		Expanded Criteria (DSM-IV)		Autism (DSM-IV)		CFR	
	P	N	P	N	P	N	P	N	P	N
Brain and Other Nervous System	1	45	1	31	1	19	1	27	1	15
Colon and Rectum	1	46	1	31	1	19	1	27	1	16
Esophagus	1	46	1	31	1	19	1	27	1	16
Hodgkin Lymphoma	1	39	1	24	1	17	1	22	1	14
Kaposi Sarcoma	1	14	1	9	1	7	1	7	1	5
Kidney and Renal Pelvis	1	46	1	31	1	19	1	27	1	16
Larynx	1	44	1	28	1	19	1	25	1	16
Leukemias	1	46	1	31	1	19	1	27	1	16
Liver and Intrahepatic Bile Duct	1	44	1	28	1	19	1	25	1	16
Lung and Bronchus	1	46	1	31	1	19	1	27	1	16
Melanomas of the Skin	1	46	1	31	1	19	1	27	1	16
Mesothelioma	1	31	1	20	1	13	1	18	1	11
Myeloma	1	44	1	29	1	19	1	26	1	16
Non-Hodgkin Lymphoma	0.983	46	0.9999	31	1	19	1	27	1	16
Oral Cavity and Pharynx	1	46	1	31	1	19	1	27	1	16
Pancreas	1	46	1	31	1	19	1	27	1	16
Prostate	1	46	1	31	1	19	1	27	1	16
Stomach	1	46	1	30	1	19	0.9944	27	1	16
Testis	1	44	1	30	1	19	1	26	1	15
Thyroid	1	42	1	27	1	18	1	24	1	15
Urinary Bladder	**0.002**	46	1	31	1	19	1	27	1	16

Pairwise correlations were performed, as described in Table 1, between state-level annual incidence for specific male cancers and autism prevalence (ages 3–21) from states selected on the basis of their criteria for diagnosing autism (Fig. 2). P represents combined *p*-values for Spearman correlations using Brown's method and bolded if P≤0.01. N represents the median number of states for which both autism and cancer data were available for analyses. Data are similar when the Pearson Correlation Coefficient is used (Table S4).

**Table 6:** Correlations between the Annual Incidence of Specific Male Adult Cancers and Autism Prevalence Subdivided by Method of Diagnosis, using Simes' P-value Method.

	ALL		Expanded Criteria (CFR)		Expanded Criteria (DSM-IV)		Autism (DSM-IV)		CFR	
	P	N	P	N	P	N	P	N	P	N
Brain and Other Nervous System	0.175	45	0.381	31	0.210	19	0.832	27	0.803	15
Colon and Rectum	0.807	46	0.996	31	0.875	19	0.484	27	0.970	16
Esophagus	0.111	46	0.277	31	0.022	19	0.694	27	0.578	16
Hodgkin Lymphoma	0.044	39	0.022	24	**0.006**	17	0.253	22	0.087	14
Kaposi Sarcoma	0.992	14	0.756	9	0.152	7	0.931	7	0.407	5
Kidney and Renal Pelvis	0.374	46	0.414	31	0.710	19	0.878	27	0.967	16
Larynx	0.487	44	0.800	28	0.633	19	0.914	25	0.866	16
Leukemias	0.996	46	0.982	31	0.991	19	0.995	27	0.924	16
Liver and Intrahepatic Bile Duct	0.272	44	0.140	28	0.991	19	0.127	25	0.852	16
Lung and Bronchus	0.984	46	0.992	31	0.994	19	0.939	27	0.824	16
Melanomas of the Skin	0.123	46	0.597	31	0.999	19	0.227	27	0.165	16
Mesothelioma	0.013	31	0.066	20	**0.006**	13	0.188	18	0.053	11
Myeloma	0.963	44	0.895	29	0.737	19	0.994	26	0.731	16
Non-Hodgkin Lymphoma	0.013	46	0.017	31	0.047	19	0.039	27	0.159	16
Oral Cavity and Pharynx	0.553	46	0.978	31	0.662	19	0.287	27	0.196	16
Pancreas	0.132	46	0.378	31	0.980	19	0.028	27	0.024	16
Prostate	0.381	46	0.848	31	0.395	19	0.999	27	0.809	16
Stomach	0.073	46	0.367	30	0.839	19	**0.004**	27	**0.008**	16
Testis	0.222	44	0.310	30	0.189	19	0.899	26	0.553	15
Thyroid	0.939	42	0.985	27	0.990	18	0.404	24	0.429	15
Urinary Bladder	**0.003**	46	0.043	31	0.030	19	0.018	27	**0.006**	16

Pairwise correlations were performed, as described in Table 1, between state-level annual incidence for specific male cancers and autism prevalence (ages 3–21) from states selected on the basis of their criteria for diagnosing autism (Fig. 2). P represents combined *p*-values for Spearman correlations using Simes' method and bolded if P≤0.01. N represents the median number of states for which both autism and cancer data were available for analyses. Data are similar when the Pearson Correlation Coefficient is used (Table S5).

## Discussion

This study utilizes information from the IDEA and CDC database that may suggest shared risk factors between autism and specific cancers. Since both the autism and cancer database contain information for up to 50 states and the District of Columbia, the sample number for conducting correlations is high, representing a potentially useful resource for these preliminary ecological analyses. However, the utility of these analyses rests on the quality of the IDEA database.

One potential limitation is that of diagnostic substitution [14], in which cases previously categorized as learning disabled or mental retardation in the 1990s may actually have been cases of autism. Although this may not be a problem in many states [15], autism as a separate category in the CFR did not occur until 1991. Another issue is that prevalence data before the age of 6 was not reported until the year 2000, probably reflecting continued refinement of the criteria for autism up to the year 2000. Our strategy to minimize this pitfall was to consider autism data only from the year 2000 forward in an effort to limit inaccurate counts due to diagnostic substitution and the changing definition of autism.

Perhaps the major criticism of the IDEA database concerns the wide range in the actual prevalence of autism in different states. As much as an eight-fold difference in autism prevalence rates has been reported between states [16]. Some states have been singled out for having unorthodox criteria (Oregon) [17], exceedingly high rates (Minnesota), a sudden 400% rise in rates from 2001–2002 (Massachusetts) [8], [18], or idiosyncratic results (California) [18]. A recent systematic study of the methods that states use to categorize autism does clarify these findings and may be helpful in extracting useful information from the IDEA database [8].

States are free to choose criteria for categorizing children with autism. School administrators and practitioners are not required to use the DSM-IV-TR to classify and to diagnose children, but they must use the diagnostic criteria outlined in the Code of Federal Regulations (CFR). Both the CFR and DSM-IV-TR recognize social interaction and communication as well as restrictive, repetitive, and stereotypical behavior, and thus the basic criteria for diagnosis are highly similar. However, the main difference between CFR and DSM-IV-TR is whether the child is disabled as a result of this diagnosis in order to qualify for special education under the autism category. Accordingly, the IDEA database underestimates autism prevalence, since it uses educational criteria for determining disability; high functioning individuals with autism who do not require special education are not counted [8].

Although states are free to choose their own eligibility criteria for special education services, they must do so as long as it meets or exceeds CFR guidelines. The legal code of every state and the District of Columbia were analyzed, along with inter-state variability [8]. As shown in Fig. 2, 17 states and the District of Columbia strictly abide by criteria used in the CFR. Interestingly, diagnosis using the CFR theme displayed high inter-rater reliability [8], and one could consider this category to represent a subset of autism as defined by DSM-IV-TR. The remaining 33 states expanded upon CFR criteria. Since the guidelines used in the CFR fall within those specified by DSM-IV-TR, states that abide by DSM-IV-TR include all those that use CFR plus an additional 13 states (Fig. 2). Autism Spectrum Disorder (ASD) includes other disorders related to autism, including Asperger's Syndrome. These “milder” disorders can account for up to 75% of the cases in some states, thus contributing greatly to the varied prevalence rates from state to state [19].

An understanding of the different criteria that states use to classify children who qualify for special education under the category of autism greatly clarifies the findings made by previous researchers who have delved into this database. For instance, all the states for which unusual or high prevalence rates were cited (Oregon, Minnesota, Massachusetts, California) are states that have expanded the eligibility criteria for autism beyond CFR. Indeed, states that have expanded their criteria beyond CFR report substantially higher prevalence rates for autism [8]. Therefore, restricting the correlation analyses to those states that adhere strictly to wording used in the CFR would represent the most conservative way to use the IDEA database, even at the cost of reducing the sample size to about a third the number of states. The second least restrictive way is to use data from states that apply DSM-IV-TR to diagnose autism, but not autism spectrum disorder.

Data were analyzed using four subdivisions of states according to criteria used to diagnose children for eligibility for special education (Fig. 2). In evaluating significance, the Bonferroni's method for correcting *p*-values due to multiple comparisons is considered very conservative, because it raises Type II error (rejection of a true correlation) while reducing Type I error [9]. Two methods for calculating an overall *p*-value based on multiple Bonferroni-adjusted *p*-values were used. Brown's method [12], which is a modification of Fisher's original inverse chi-square method [11], takes all *p*-values into account and determines if the log transformation of all the values fall within a chi-square distribution. Thus, multiple *p*-values need to show significance before the overall *p*-value becomes significant; a single *p*-value, even if very significant, will not result in an overall significance. A less conservative method, the Simes' procedure [13], determines if at least one *p*-value out of a set of *p*-values is significant. As can be observed from Tables 3–6, a few correlations meet significance using these conditions.

When the most restrictive criteria for selecting state-level autism data were used, states abiding by CFR strictly, and Brown's method for combining Bonferroni-adjusted *p*-values applied, only one correlation was significant: the correlation between autism prevalence and the incidence of *in situ* breast cancer (*p*<10^−10^; N = 16). When a less conservative statistical method was applied (Simes' procedure), correlations between autism and uterine cancer also emerged as consistently significant. By contrast, the great majority of correlations between specific forms of cancer and autism were negative. Although Type II error may have been increased as a result of these methods, it is appropriate in light of the controversial use of the IDEA database.

In conclusion, by using conservative statistical methods and a limited set of autism data from states using a uniform code of diagnosis, nominal statistical significance was observed in a few instances, notably for breast cancer and uterine cancer. In practice, it is not known whether the diagnosis of autism is truly uniform in individual school districts. Consequently, the results should be interpreted with caution, even if the *p*-values appear to be selective for these cancers and highly significant, as is the case here. Nonetheless, it is of interest that the cumulative exposure to estrogen from endogenous and external sources is an established risk factor for both breast [20] and uterine [21] cancer, the two cancers that appear to be most consistently correlated with autism. Some analyses suggest that mothers are carriers of mutations that predispose children to autism [22], and there is literature implicating germline mutations in autism [23], [24]. In this context, we suggest that investigating biomedical mechanisms to account for these epidemiological findings is warranted.

## Materials and Methods

### Sources of Data

The number of children diagnosed with autism was collected for all states and ages between the years 2000–2007 from the U.S. Department of Education via the Individuals with Disabilities Act (IDEA) (https://www.ideadata.org). Six age groups were analyzed: 3–5, 6–8, 9–11, 12–14, 15–17, and 18–20 years; as well as the entire span of ages, 3–21. Autism prevalence separated by gender or before the age of 3 are not available. Annual resident population numbers by age and respective year (2000–2007) were obtained from the U.S. Census Bureau (http://www.census.gov), and used as the denominator to calculate the annual prevalence of autism in each state.

The age-adjusted annual incidence of specific cancers (standardized to the 2000 U.S. population) for males and females and for all states between the years 1999 and 2005 were obtained from the CDC (http://apps.nccd.cdc.gov/uscs/), the years presently available.

### Statistical Analyses

The Spearman rank correlation coefficients were calculated by comparing the prevalence of autism to the annual incidence of cancer, at the state level, throughout the U.S. This was done for each autism age group and year reported, and for each type of cancer and year reported. Significance was calculated using methods previously described [25], and adjusted using the Bonferroni correction [9], [10].

To obtain an overall significance or combined *p*-value for each set of correlations, three methods were used and compared: Fisher's inverse chi-square method [11], Brown's method for combining dependent *p*-values [12] and Simes' procedure [13].

## Supporting Information

Table S1 Correlations Between the Annual Incidence of All Adult Cancers Combined and Autism Prevalence Subdivided by Method of Diagnosis. Pairwise correlations were performed as described in Table 1 using two methods, Brown and Simes [12], [13], for combining dependent p-values. Autism prevalence data (ages 3–21) were obtained from groups of states selected on the basis of their criteria for diagnosing autism in all states or states subdivided by 4 groups of criteria (Fig. 2). P represents combined p-values for the Pearson correlations and bolded if P≤0.01. N represents the median number of states for which both autism and cancer data were available for analyses.
(0.03 MB DOC)

Table S2 Correlations Between the Annual Incidence of Specific Female Adult Cancers and Autism Prevalence Subdivided by Method of Diagnosis, using Brown's P-value Method. Pairwise correlations were performed, as described in Table 1, between state-level annual incidence for specific female cancers and autism prevalence (ages 3–21) from states selected on the basis of their criteria for diagnosing autism (Fig. 2). P represents combined p-values for Pearson correlations using Brown's method and bolded if P≤0.01. N represents the median number of states for which both autism and cancer data were available for analyses. Kaposi's sarcoma is omitted because there was insufficient data to conduct the analyses.
(0.06 MB DOC)

Table S3 Correlations Between the Annual Incidence of Specific Female Adult Cancers and Autism Prevalence Subdivided by Method of Diagnosis, using Simes' P-value Method. Pairwise correlations were performed, as described in Table 1, between state-level annual incidence for specific female cancers and autism prevalence (ages 3–21) from states selected on the basis of their criteria for diagnosing autism (Fig. 2). P represents combined p-values for Pearson correlations using Simes' method and bolded if P≤0.01. N represents the median number of states for which both autism and cancer data were available for analyses. Kaposi's sarcoma is omitted because there was insufficient data to conduct the analyses.
(0.05 MB DOC)

Table S4 Correlations Between the Annual Incidence of Specific Male Adult Cancers and Autism Prevalence Subdivided by Method of Diagnosis, using Brown's P-value Method. Pairwise correlations were performed, as described in Table 1, between state-level annual incidence for specific male cancers and autism prevalence (ages 3–21) from states selected on the basis of their criteria for diagnosing autism (Fig. 2). P represents combined p-values for Pearson correlations using Brown's method and bolded if P≤0.01. N represents the median number of states for which both autism and cancer data were available for analyses.
(0.05 MB DOC)

Table S5 Correlations Between the Annual Incidence of Specific Male Adult Cancers and Autism Prevalence Subdivided by Method of Diagnosis, using Simes' P-value Method. Pairwise correlations were performed, as described in Table 1, between state-level annual incidence for specific male cancers and autism prevalence (ages 3–21) from states selected on the basis of their criteria for diagnosing autism (Fig. 2). P represents combined p-values for Pearson correlations using Simes' method and bolded if P≤0.01. N represents the median number of states for which both autism and cancer data were available for analyses.
(0.05 MB DOC)

## References

[1] Newschaffer CJ, Curran LK (2003). Autism: an emerging public health problem.. Public Health Rep.

[2] Vorstman JA, Staal WG, van Daalen E, van Engeland H, Hochstenbach PF (2006). Identification of novel autism candidate regions through analysis of reported cytogenetic abnormalities associated with autism.. Mol Psychiatry.

[3] Christian SL, Brune CW, Sudi J, Kumar RA, Liu S (2008). Novel submicroscopic chromosomal abnormalities detected in autism spectrum disorder.. Biol Psychiatry.

[4] Marshall CR, Noor A, Vincent JB, Lionel AC, Feuk L (2008). Structural variation of chromosomes in autism spectrum disorder.. Am J Hum Genet.

[5] Morrow EM, Yoo SY, Flavell SW, Kim TK, Lin Y (2008). Identifying autism loci and genes by tracing recent shared ancestry.. Science.

[6] Sebat J, Lakshmi B, Malhotra D, Troge J, Lese-Martin C (2007). Strong association of de novo copy number mutations with autism.. Science.

[7] Szatmari P, Paterson AD, Zwaigenbaum L, Roberts W, Brian J (2007). Mapping autism risk loci using genetic linkage and chromosomal rearrangements.. Nat Genet.

[8] MacFarlane JR, Kanaya T (2009). What does it mean to be autistic? Inter-state variation in special education criteria for autism services.. J Child Fam Stud.

[9] Curtin F, Schulz P (1998). Multiple correlations and Bonferroni's correction.. Biol Psychiatry.

[10] Wright SP (1992). Adjusted p-values for simultaneous inference.. Biometrics.

[11] Fisher RA (1932). Statistical Methods for Research Workers.

[12] Brown MB (1975). A method for combining non-independent, one-sided tests of significance.. Biometrics.

[13] Simes R (1986). An improved Bonferroni procedure for multiple tests of significance.. Biometrika.

[14] Shattuck PT (2006). Diagnostic substitution and changing autism prevalence.. Pediatrics.

[15] Newschaffer CJ, Falb MD, Gurney JG (2005). National autism prevalence trends from United States special education data.. Pediatrics.

[16] Mandell DS, Palmer R (2005). Differences among states in the identification of autistic spectrum disorders.. Arch Pediatr Adolesc Med.

[17] Laidler JR (2005). US Department of Education data on “autism” are not reliable for tracking autism prevalence.. Pediatrics.

[18] Gernsbacher MA, Dawson M, Goldsmith HH (2005). Three reasons not to believe in an autism epidemic.. Curr Dir Psychol Sci.

[19] Chakrabarti S, Fombonne E (2001). Pervasive developmental disorders in preschool children.. Jama.

[20] Yager JD, Davidson NE (2006). Estrogen carcinogenesis in breast cancer.. N Engl J Med.

[21] Amant F, Moerman P, Neven P, Timmerman D, Van Limbergen E (2005). Endometrial cancer.. Lancet.

[22] Zhao X, Leotta A, Kustanovich V, Lajonchere C, Geschwind DH (2007). A unified genetic theory for sporadic and inherited autism.. Proc Natl Acad Sci U S A.

[23] Reichenberg A, Gross R, Weiser M, Bresnahan M, Silverman J (2006). Advancing paternal age and autism.. Arch Gen Psychiatry.

[24] Durkin MS, Maenner MJ, Newschaffer CJ, Lee LC, Cunniff CM (2008). Advanced parental age and the risk of autism spectrum disorder.. Am J Epidemiol.

[25] Zar JH (1972). Significance testing of the Spearman rank correlation coefficient.. J Am Stat Assoc.

