# Supplementary material for: The Correlation between Rates of Cancer and Autism: An Exploratory Ecological Investigation
Source: PLoS One. 2010 Feb 23;5(2):e9372. doi: 10.1371/journal.pone.0009372 (PMC2826417; doi:10.1371/journal.pone.0009372)
Supplement: Table S1 — Correlations Between the Annual Incidence of All Adult Cancers Combined and Autism Prevalence Subdivided by Method of Diagnosis. Pairwise correlations were performed as described in Table 1 using two methods, Brown and Simes [12], [13], for combining dependent p-values. Autism prevalence data (ages 3–21) were obtained from groups of states selected on the basis of their criteria for diagnosing autism in all states or states subdivided by 4 groups of criteria (Fig. 2). P represents combined p-values for the Pearson correlations and bolded if P≤0.01. N represents the median number of states for which both autism and cancer data were available for analyses. (0.03 MB DOC) [file pone.0009372.s001.doc]

Table S1. Correlations Between the Annual Incidence of All Adult Cancers Combined and Autism Prevalence Subdivided by Method of Diagnosis.

|  |  | **ALL** | | **Expanded Criteria (CFR)** | | **Expanded Criteria (DSM-IV)** | | **Autism (DSM-IV)** | | **CFR** | |
| --- | --- | --- | --- | --- | --- | --- | --- | --- | --- | --- | --- |
|  | Method for Combining *p*-values | **P** | **N** | **P** | **N** | **P** | **N** | **P** | **N** | **P** | **N** |
| **All Female Cancers** | Brown | 0.154 | 46 | 1 | 31 | 1 | 19 | 1 | 27 | 1 | 16 |
|  | Simes | **0.006** | 46 | 0.015 | 31 | 0.083 | 19 | 0.048 | 27 | 0.113 | 16 |
| **All Male Cancers** | Brown | 1 | 46 | 1 | 32 | 1 | 19 | 1 | 27 | 1 | 16 |
|  | Simes | 0.343 | 46 | 0.462 | 32 | 0.647 | 19 | 0.674 | 27 | 0.682 | 16 |

Pairwise correlations were performed as described in Table 1 using two methods, Brown and Simes [12,13], for combining dependent *p*-values. Autism prevalence data (ages 3-21) were obtained from groups of states selected on the basis of their criteria for diagnosing autism in all states or states subdivided by 4 groups of criteria (Fig. 2). P represents combined *p*-values for the Pearson correlations and bolded if P≤0.01. N represents the median number of states for which both autism and cancer data were available for analyses.
