# Supplementary material for: The Correlation between Rates of Cancer and Autism: An Exploratory Ecological Investigation
Source: PLoS One. 2010 Feb 23;5(2):e9372. doi: 10.1371/journal.pone.0009372 (PMC2826417; doi:10.1371/journal.pone.0009372)
Supplement: Table S3 — Correlations Between the Annual Incidence of Specific Female Adult Cancers and Autism Prevalence Subdivided by Method of Diagnosis, using Simes' P-value Method. Pairwise correlations were performed, as described in Table 1, between state-level annual incidence for specific female cancers and autism prevalence (ages 3–21) from states selected on the basis of their criteria for diagnosing autism (Fig. 2). P represents combined p-values for Pearson correlations using Simes' method and bolded if P≤0.01. N represents the median number of states for which both autism and cancer data were available for analyses. Kaposi's sarcoma is omitted because there was insufficient data to conduct the analyses. (0.05 MB DOC) [file pone.0009372.s003.doc]

Table S3. Correlations Between the Annual Incidence of Specific Female Adult Cancers and Autism Prevalence Subdivided by Method of Diagnosis, using Simes’ P-value Method.

|  | **ALL** | | **Expanded Criteria (CFR)** | | **Expanded Criteria (DSM-IV)** | | **Autism (DSM-IV)** | | **CFR** | |
| --- | --- | --- | --- | --- | --- | --- | --- | --- | --- | --- |
|  | **P** | **N** | **P** | **N** | **P** | **N** | **P** | **N** | **P** | **N** |
| Brain and Other Nervous System | 0.752 | 44 | 0.920 | 29 | 0.996 | 19 | 1.000 | 25 | 0.918 | 15 |
| Breast, invasive | **0.003** | 46 | **0.004** | 30 | **0.004** | 19 | 0.043 | 27 | 0.149 | 16 |
| Breast, *in situ* | **0.002** | 46 | 0.042 | 30 | 0.214 | 19 | **0.001** | 27 | **0.001** | 16 |
| Cervix Uteri | 0.070 | 46 | 0.399 | 30 | 0.076 | 19 | 0.532 | 27 | 0.028 | 16 |
| Colon and Rectum | 0.818 | 46 | 0.981 | 30 | 0.974 | 19 | 0.365 | 27 | 0.401 | 16 |
| Corpus and Uterus, NOS | **0.007** | 46 | 0.065 | 30 | 0.165 | 19 | **0.003** | 27 | **0.005** | 16 |
| Esophagus | 0.093 | 34 | 0.442 | 22 | 0.573 | 15 | 0.024 | 20 | **0.0004** | 13 |
| Hodgkin Lymphoma | 0.022 | 37 | 0.098 | 24 | 0.432 | 16 | 0.023 | 21 | 0.049 | 14 |
| Kidney and Renal Pelvis | 0.951 | 46 | 0.877 | 30 | 0.976 | 19 | 0.994 | 27 | 0.989 | 16 |
| Larynx | 0.078 | 32 | 0.133 | 21 | 0.195 | 14 | 0.288 | 18 | 0.367 | 11 |
| Leukemias | 0.534 | 46 | 0.938 | 30 | 0.873 | 19 | 0.391 | 27 | 0.514 | 16 |
| Liver and Intrahepatic Bile Duct | 0.934 | 38 | 0.824 | 24 | 0.876 | 16 | 0.995 | 22 | 0.999 | 14 |
| Lung and Bronchus | 0.194 | 46 | 0.225 | 30 | 0.296 | 19 | 0.990 | 27 | 0.915 | 16 |
| Melanomas of the Skin | 0.064 | 46 | 0.201 | 30 | 0.281 | 19 | 0.764 | 27 | 0.401 | 16 |
| Mesothelioma | 0.343 | 13 | 0.106 | 9 | 0.035 | 6 | 0.987 | 7 | NA | 4 |
| Myeloma | 0.344 | 43 | 0.305 | 28 | 0.878 | 18 | 0.973 | 25 | 0.993 | 16 |
| Non-Hodgkin Lymphoma | **0.005** | 46 | **0.006** | 30 | 0.046 | 19 | 0.245 | 27 | 0.566 | 16 |
| Oral Cavity and Pharynx | 0.102 | 45 | 0.114 | 29 | 0.502 | 19 | 0.067 | 26 | 0.506 | 16 |
| Ovary | 0.571 | 46 | 0.921 | 30 | 0.670 | 19 | 0.793 | 27 | 0.729 | 16 |
| Pancreas | 0.260 | 46 | 0.284 | 30 | 0.969 | 19 | **0.006** | 27 | 0.094 | 16 |
| Stomach | 0.571 | 43 | 0.817 | 27 | 0.698 | 18 | 0.125 | 25 | 0.347 | 16 |
| Thyroid | 0.993 | 46 | 0.882 | 30 | 0.979 | 19 | 0.697 | 27 | 0.543 | 16 |
| Urinary Bladder | **0.008** | 46 | 0.050 | 30 | 0.151 | 19 | 0.081 | 27 | 0.023 | 16 |

Pairwise correlations were performed, as described in Table 1, between state-level annual incidence for specific female cancers and autism prevalence (ages 3-21) from states selected on the basis of their criteria for diagnosing autism (Fig. 2). P represents combined *p*-values for Pearson correlations using Simes' method and bolded if P≤0.01. N represents the median number of states for which both autism and cancer data were available for analyses. Kaposi’s sarcoma is omitted because there was insufficient data to conduct the analyses.
