# Supplementary material for: The Correlation between Rates of Cancer and Autism: An Exploratory Ecological Investigation
Source: PLoS One. 2010 Feb 23;5(2):e9372. doi: 10.1371/journal.pone.0009372 (PMC2826417; doi:10.1371/journal.pone.0009372)
Supplement: Table S4 — Correlations Between the Annual Incidence of Specific Male Adult Cancers and Autism Prevalence Subdivided by Method of Diagnosis, using Brown's P-value Method. Pairwise correlations were performed, as described in Table 1, between state-level annual incidence for specific male cancers and autism prevalence (ages 3–21) from states selected on the basis of their criteria for diagnosing autism (Fig. 2). P represents combined p-values for Pearson correlations using Brown's method and bolded if P≤0.01. N represents the median number of states for which both autism and cancer data were available for analyses. (0.05 MB DOC) [file pone.0009372.s004.doc]

Table S4. Correlations Between the Annual Incidence of Specific Male Adult Cancers and Autism Prevalence Subdivided by Method of Diagnosis, using Brown's P-value Method.

|  | **ALL** | | **Expanded Criteria (CFR)** | | **Expanded Criteria (DSM-IV)** | | **Autism (DSM-IV)** | | **CFR** | |
| --- | --- | --- | --- | --- | --- | --- | --- | --- | --- | --- |
|  | **P** | **N** | **P** | **N** | **P** | **N** | **P** | **N** | **P** | **N** |
| Brain and Other Nervous System | 1 | 45 | 1 | 31 | 1 | 19 | 1 | 27 | 1 | 15 |
| Colon and Rectum | 1 | 46 | 1 | 31 | 1 | 19 | 1 | 27 | 1 | 16 |
| Esophagus | 1 | 46 | 1 | 31 | 1 | 19 | 1 | 27 | 1 | 16 |
| Hodgkin Lymphoma | 1 | 39 | 1 | 24 | 1 | 17 | 1 | 22 | 1 | 14 |
| Kaposi Sarcoma | 1 | 14 | 1 | 9 | 1 | 7 | 1 | 7 | 1 | 5 |
| Kidney and Renal Pelvis | 1 | 46 | 1 | 31 | 1 | 19 | 1 | 27 | 1 | 16 |
| Larynx | 1 | 44 | 1 | 28 | 1 | 19 | 1 | 25 | 1 | 16 |
| Leukemias | 1 | 46 | 1 | 31 | 1 | 19 | 1 | 27 | 1 | 16 |
| Liver and Intrahepatic Bile Duct | 1 | 44 | 1 | 28 | 1 | 19 | 1 | 25 | 1 | 16 |
| Lung and Bronchus | 1 | 46 | 1 | 31 | 1 | 19 | 1 | 27 | 1 | 16 |
| Melanomas of the Skin | 1 | 46 | 1 | 31 | 1 | 19 | 1 | 27 | 1 | 16 |
| Mesothelioma | 1 | 31 | 1 | 20 | 1 | 13 | 1 | 18 | 1 | 11 |
| Myeloma | 1 | 44 | 1 | 29 | 1 | 19 | 1 | 26 | 1 | 16 |
| Non-Hodgkin Lymphoma | 0.843 | 46 | 0.966 | 31 | 1 | 19 | 1 | 27 | 1 | 16 |
| Oral Cavity and Pharynx | 1 | 46 | 1 | 31 | 1 | 19 | 1 | 27 | 1 | 16 |
| Pancreas | 1 | 46 | 1 | 31 | 1 | 19 | 1 | 27 | 1 | 16 |
| Prostate | 1 | 46 | 1 | 31 | 1 | 19 | 1 | 27 | 1 | 16 |
| Stomach | 1 | 46 | 1 | 30 | 1 | 19 | 0.991 | 27 | 1 | 16 |
| Testis | 1 | 44 | 1 | 30 | 1 | 19 | 1 | 26 | 1 | 15 |
| Thyroid | 1 | 42 | 1 | 27 | 1 | 18 | 1 | 24 | 1 | 15 |
| Urinary Bladder | 0.994 | 46 | 1 | 31 | 1 | 19 | 1 | 27 | 0.999 | 16 |

Pairwise correlations were performed, as described in Table 1, between state-level annual incidence for specific male cancers and autism prevalence (ages 3-21) from states selected on the basis of their criteria for diagnosing autism (Fig. 2). P represents combined *p*-values for Pearson correlations using Brown’s method and bolded if P≤0.01. N represents the median number of states for which both autism and cancer data were available for analyses.
