# Supplementary material for: The Correlation between Rates of Cancer and Autism: An Exploratory Ecological Investigation
Source: PLoS One. 2010 Feb 23;5(2):e9372. doi: 10.1371/journal.pone.0009372 (PMC2826417; doi:10.1371/journal.pone.0009372)
Supplement: Table S5 — Correlations Between the Annual Incidence of Specific Male Adult Cancers and Autism Prevalence Subdivided by Method of Diagnosis, using Simes' P-value Method. Pairwise correlations were performed, as described in Table 1, between state-level annual incidence for specific male cancers and autism prevalence (ages 3–21) from states selected on the basis of their criteria for diagnosing autism (Fig. 2). P represents combined p-values for Pearson correlations using Simes' method and bolded if P≤0.01. N represents the median number of states for which both autism and cancer data were available for analyses. (0.05 MB DOC) [file pone.0009372.s005.doc]

Table S5. Correlations Between the Annual Incidence of Specific Male Adult Cancers and Autism Prevalence Subdivided by Method of Diagnosis, using Simes' P-value Method.

|  | **ALL** | | **Expanded Criteria (CFR)** | | **Expanded Criteria (DSM-IV)** | | **Autism (DSM-IV)** | | **CFR** | |
| --- | --- | --- | --- | --- | --- | --- | --- | --- | --- | --- |
|  | **P** | **N** | **P** | **N** | **P** | **N** | **P** | **N** | **P** | **N** |
| Brain and Other Nervous System | 0.039 | 45 | 0.112 | 31 | 0.156 | 19 | 0.845 | 27 | 0.678 | 15 |
| Colon and Rectum | 0.989 | 46 | 0.986 | 31 | 0.991 | 19 | 0.354 | 27 | 0.660 | 16 |
| Esophagus | 0.137 | 46 | 0.394 | 31 | 0.284 | 19 | 0.664 | 27 | 0.440 | 16 |
| Hodgkin Lymphoma | 0.116 | 39 | 0.091 | 24 | 0.152 | 17 | 0.241 | 22 | 0.056 | 14 |
| Kaposi Sarcoma | 0.972 | 14 | 0.795 | 9 | 0.308 | 7 | 0.846 | 7 | 0.437 | 5 |
| Kidney and Renal Pelvis | 0.767 | 46 | 0.902 | 31 | 0.997 | 19 | 0.851 | 27 | 0.999 | 16 |
| Larynx | 0.358 | 44 | 0.360 | 28 | 0.222 | 19 | 0.968 | 25 | 0.971 | 16 |
| Leukemias | 0.969 | 46 | 0.996 | 31 | 0.978 | 19 | 0.990 | 27 | 0.897 | 16 |
| Liver and Intrahepatic Bile Duct | 0.945 | 44 | 0.957 | 28 | 0.977 | 19 | 0.138 | 25 | 0.921 | 16 |
| Lung and Bronchus | 0.924 | 46 | 0.998 | 31 | 0.723 | 19 | 0.863 | 27 | 0.888 | 16 |
| Melanomas of the Skin | 0.180 | 46 | 0.487 | 31 | 0.866 | 19 | 0.197 | 27 | 0.211 | 16 |
| Mesothelioma | 0.016 | 31 | 0.020 | 20 | 0.024 | 13 | 0.159 | 18 | 0.049 | 11 |
| Myeloma | 0.854 | 44 | 0.862 | 29 | 0.099 | 19 | 0.950 | 26 | 0.975 | 16 |
| Non-Hodgkin Lymphoma | 0.008 | 46 | **0.006** | 31 | 0.016 | 19 | 0.016 | 27 | 0.072 | 16 |
| Oral Cavity and Pharynx | 0.989 | 46 | 0.995 | 31 | 0.797 | 19 | 0.388 | 27 | 0.397 | 16 |
| Pancreas | 0.729 | 46 | 0.970 | 31 | 0.973 | 19 | 0.026 | 27 | 0.059 | 16 |
| Prostate | 0.445 | 46 | 0.950 | 31 | 0.384 | 19 | 0.980 | 27 | 0.934 | 16 |
| Stomach | 0.785 | 46 | 0.918 | 30 | 0.990 | 19 | **0.005** | 27 | 0.029 | 16 |
| Testis | 0.081 | 44 | 0.138 | 30 | 0.070 | 19 | 0.646 | 26 | 0.950 | 15 |
| Thyroid | 0.837 | 42 | 0.888 | 27 | 0.880 | 18 | 0.393 | 24 | 0.351 | 15 |
| Urinary Bladder | **0.010** | 46 | 0.076 | 31 | 0.239 | 19 | 0.032 | 27 | **0.006** | 16 |

Pairwise correlations were performed, as described in Table 1, between state-level annual incidence for specific male cancers and autism prevalence (ages 3-21) from states selected on the basis of their criteria for diagnosing autism (Fig. 2). P represents combined *p*-values for Pearson correlations using Simes’ method and bolded if P≤0.01. N represents the median number of states for which both autism and cancer data were available for analyses.
